# Developing and validating a regulatory science competency framework for immune cell therapy: a mixed-methods study

**DOI:** 10.3389/fmed.2026.1917978

**Published:** 2026-09-16

**Authors:** Jung-A Lee

**Affiliations:** College of Pharmacy, Seoul National University, Seoul, Republic of Korea

**Keywords:** adaptive expertise, advanced therapy medicinal products, competency framework, competency-based education, Delphi method, immune cell therapy, mixed-methods research, regulatory science

## Abstract

**Background:**

Advanced therapy medicinal products (ATMPs), particularly immune cell therapies, present distinctive regulatory challenges due to biological variability, evolving manufacturing processes, and uncertainty throughout the product lifecycle. Existing competency frameworks for regulatory science are predominantly generalist and may not adequately capture the domain-specific competencies required for immune cell therapy.

**Objective:**

To develop and validate a consensus-based competency framework for regulatory science professionals in immune cell therapy.

**Methods:**

An exploratory sequential mixed-methods design was employed, comprising a multi-source literature review, preliminary expert consultation, semi-structured expert interviews, and a two-round Delphi consensus process. Experts representing academia, industry, regulatory authorities, and government/public-sector organizations participated in the preliminary consultation and interviews (*n* = 11) and the Delphi consensus process (*n* = 20). Qualitative data were analyzed using reflexive thematic analysis. Delphi responses were analyzed using descriptive statistics, the content validity ratio, and the item-level content validity index to assess content validity in Round 1, and retention agreement, importance, educational applicability, and measurability in Round 2.

**Results:**

The final framework comprised nine core competencies and 32 sub-competencies organized into three areas: Scientific and Regulatory Foundations, Strategic Application of Regulatory Science, and Regulatory Leadership and Responsibility. High retention agreement (95%–100%) indicated the stability of the framework. Sub-competencies related to leadership, ethics, and higher-order judgment received relatively lower ratings for educational applicability and measurability but were retained because of their conceptual importance.

**Conclusion:**

This study presents a domain-specific competency framework that conceptualizes regulatory competence in immune cell therapy as an integrative and adaptive capability. The framework provides a practical foundation for competency-based training, assessment, and regulatory science workforce development in immune cell therapy and related ATMPs.

## 1 Introduction

Advanced therapy medicinal products (ATMPs) have fundamentally transformed the treatment landscape for a range of previously intractable diseases, offering new possibilities for long-term remission or even cure (1, 2). ATMPs encompass gene therapies, cell therapies, and tissue-engineered products, and are increasingly central to national strategies for regenerative medicine and advanced biologics (3–5). Among these, immune cell therapies (e.g., CAR-T, TCR-T, and TIL therapies) represent a rapidly expanding domain that has transformed the treatment of hematologic malignancies and is increasingly being explored for solid tumors (6, 7).

These immune cell therapies, often described as “living drugs,” offer unprecedented therapeutic potential but also introduce profound regulatory challenges due to inherent biological variability, evolving manufacturing processes, and the dynamic nature of living cell products (8–11). Unlike traditional small-molecule drugs or biologics, many ATMPs do not conform to stable and reproducible product paradigms. This challenge is particularly pronounced in immune cell therapies, where product characteristics and manufacturing processes may evolve throughout development (1, 12, 13).

Consequently, regulatory science professionals working in ATMPs must move beyond technical assessments of safety, efficacy, and quality to exercise integrative and context-sensitive judgment under conditions of uncertainty (2, 12). This challenge is global but particularly acute in rapidly evolving regulatory environments such as South Korea, where regulatory activity accelerated following the enactment of the Act on the Safety and Support of Advanced Regenerative Medicine and Advanced Biological Products in 2020 (14, 15). Increased clinical trial activity, approvals of CAR-T therapies (e.g., tisagenlecleucel and axicabtagene ciloleucel), and updated Ministry of Food and Drug Safety (MFDS) guidelines highlight the growing demand for a specialized regulatory science workforce (16–18). Despite this progress, the scientific, manufacturing, and ethical complexities inherent in ATMPs, particularly immune cell therapies, continue to challenge existing regulatory systems (13), creating a gap between emerging regulatory demands and current workforce preparedness.

Current educational and training programs in regulatory science remain largely generalist, focusing on broad biopharmaceutical processes rather than the domain-specific requirements of advanced therapies (19–21). As a result, professionals may be insufficiently prepared to navigate scientific uncertainties and engage in interdisciplinary decision-making required for advanced therapies. Existing competency frameworks in regulatory science and regulatory affairs typically conceptualize competence as discrete, task-based knowledge and skills suited to relatively stable environments (3, 22, 23). Although these frameworks provide valuable role clarification, they leave limited room for the context-sensitive judgment required in advanced therapy regulation (12).

Professional competence in health professions education has increasingly been reconceptualized as an integrative and adaptive construct rather than a simple aggregation of knowledge and skills (24). Similarly, scholarship on expertise distinguishes routine expertise from adaptive expertise. While routine expertise emphasizes the efficient application of established procedures, adaptive expertise involves the flexible application of conceptual knowledge and the generation of novel solutions in unfamiliar situations (25, 26). Competency-based medical education (CBME) builds on these perspectives by emphasizing observable performance, progression toward mastery, and contextual integration of knowledge, skills, and judgment through experiential learning and reflection (27–29).

These integrative and adaptive capabilities are particularly essential in regulatory science for ATMPs (13). International initiatives, such as the European Medicines Agency's (EMA) Regulatory Science to 2025 strategy and programs led by the U.S. Food and Drug Administration (FDA), have provided strategic guidance for regulatory science workforce development. However, these initiatives are predominantly policy-oriented and do not adequately address product-specific uncertainties or domain-specific competency requirements (3–5, 24).

Despite growing recognition of competency-based approaches and adaptive expertise in professional education, competency frameworks tailored to regulatory science for ATMPs remain underdeveloped (2, 30). Major existing frameworks, such as the World Health Organization (WHO)'s Global Competency Framework for Regulators of Medicines (23) and the Regulatory Affairs Professionals Society (RAPS)'s Regulatory Competency Framework (22), provide comprehensive role-based competencies for medicines regulators and regulatory affairs professionals. However, these frameworks are intended for broad regulatory practice and do not adequately address the product-specific uncertainties inherent in ATMPs, particularly immune cell therapies. While recent studies have begun to explore competency requirements for ATMP development and translation, these efforts have primarily emphasized translational and product development perspectives rather than regulatory science practice (2, 15, 30).

To address these gaps, this study aimed to develop a consensus-based competency framework for regulatory science professionals in immune cell therapy, a representative domain of ATMPs. The resulting framework contributes to the literature on competency development in regulatory science by providing a domain-specific, consensus-based competency framework for immune cell therapy. It may serve as a context-sensitive basis for curriculum design, competency assessment, and workforce development in immune cell therapy and other advanced therapy fields.

## 2 Methods

### 2.1 Study design

An exploratory sequential mixed-methods design was employed to develop and validate a competency framework for regulatory science professionals specializing in immune cell therapy. This design was selected to address the complexity and limited prior conceptualization of the domain by enabling an initial qualitative exploration of competency requirements, followed by a quantitative two-round Delphi consensus process to establish expert consensus on the competency framework (31). The qualitative component employed a qualitative descriptive approach to explore experts’ perspectives on competency requirements in this domain (32).

The study was informed by scholarship on professional competence, competency framework development, and competency-based education, which emphasize the identification and organization of competencies required for effective performance in complex professional contexts (27, 33, 34). These perspectives informed competency identification and framework structuring.

The study progressed through four iterative phases: (1) a multi-source literature review, (2) preliminary expert consultation, (3) semi-structured expert interviews, and (4) a two-round Delphi consensus process. Findings from each phase informed iterative refinement of the framework, culminating in final confirmation of the refined framework through Delphi-based expert consensus. Reporting of the mixed-methods study was guided by the Good Reporting of A Mixed Methods Study (GRAMMS) recommendations (35). The overall framework development process is illustrated in Figure 1.

**Figure 1:**
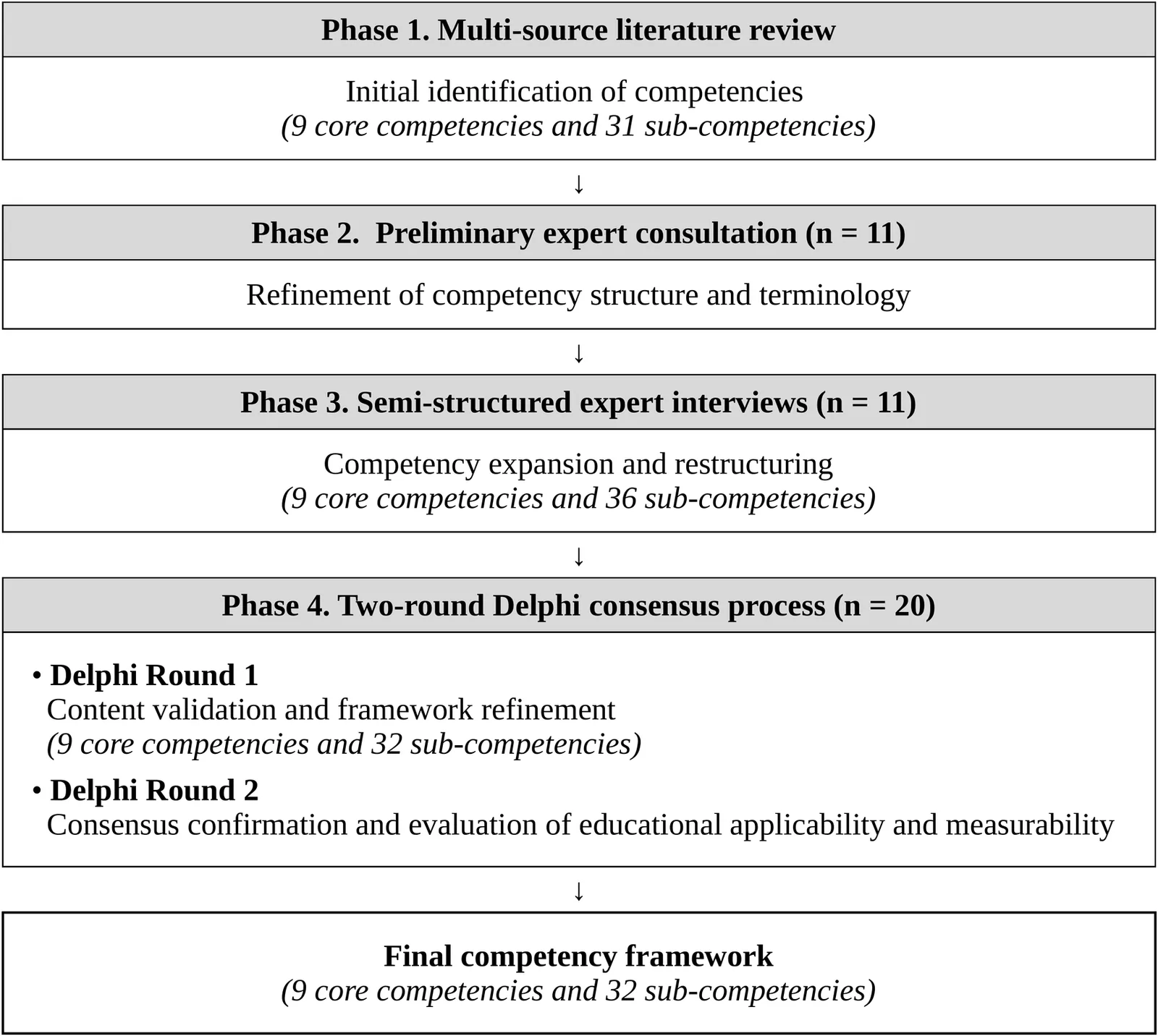
Multiphase development process for the regulatory science competency framework in immune cell therapy, showing the iterative identification, refinement, and confirmation of core competencies and sub-competencies across study phases.

### 2.2 Participants

Experts with substantial experience in immune cell therapy development and regulatory science were recruited using purposive sampling, a strategy commonly used in qualitative and mixed-methods research to identify information-rich participants aligned with the study objectives (36). This approach ensured representation across key stakeholder groups, including academia, industry, regulatory authorities, and government/public-sector organizations, reflecting the multidisciplinary nature of regulatory science in advanced therapy medicinal products.

Participants were eligible if they had at least 10 years of professional experience and direct involvement in immune cell therapy development, regulatory science, regulatory affairs, regulatory review, policy development, or health professions education. Potential participants were identified through professional networks, expert recommendations, and institutional contacts related to immune cell therapy and regulatory science.

In the qualitative phase, 11 experts participated in preliminary expert consultations and semi-structured interviews. Participants were selected based on their experience in immune cell therapy research, advanced biopharmaceutical development, regulatory affairs, regulatory review, and policy development. The sample included academic experts (*n* = 3), industry professionals involved in advanced therapy development and regulation (*n* = 5), regulatory authority representatives (*n* = 1), and government/public-sector professionals (*n* = 2), with professional experience ranging from 12 to 36 years (mean=24.0 years) (Table 1).

**Table 1:** Characteristics of expert interview participants (*N* = 11).

ID	Sector	Organization type	Primary expertise	Years of experience
P1	Academia	Medical school	Pediatric hematology and CAR-T therapy	32
P2	Academia	Pharmacy school	CAR-T engineering and translational research	16
P3	Academia	Pharmacy school	Tumor immunology and cell therapy	13
P4	Industry	Cell therapy company	Advanced therapy clinical development and commercialization	31
P5	Industry	CAR-T company	CAR-T regulatory affairs and regulatory strategy	23
P6	Industry	Cell therapy company	Cell therapy development and translational research	19
P7	Industry	Cell therapy company	Advanced therapy regulatory affairs and product approval	12
P8	Regulatory consultant	Regulatory consulting firm	Biologics regulation and regulatory strategy	29
P9	Regulatory authority	National regulatory agency	Cell and gene therapy review and regulation	23
P10	Government/ public sector	National R&D Program	Regenerative medicine policy and governance	36
P11	Government/ public sector	Government health innovation program	Advanced biopharmaceutical policy and workforce development	30

For the Delphi consensus process, a panel of 20 experts was recruited using purposive sampling to ensure representation across key stakeholder groups and a broad range of expertise relevant to immune cell therapy and regulatory science. The panel size and composition aligned with established recommendations for Delphi studies, which emphasize diverse expertise and adequate panel size for robust consensus development (37, 38). The Delphi panel consisted of experts from academia (*n* = 6), industry (*n* = 8), regulatory authorities (*n* = 2), and government/public-sector organizations (*n* = 4). The panel members had a mean of 24.5 years of professional experience. Their expertise spanned immune cell therapy research, regulatory science, and health professions education (Table 2).

**Table 2:** Characteristics of Delphi panel participants (*N* = 20).

Category	Subcategory	n	%
Gender	Male	11	55
	Female	9	45
Sector	Academia	6	30
	Industry	8	40
	Regulatory authorities	2	10
	Government/Public sector	4	20
Years of experience	≤15 years	3	15
	16–25 years	9	45
	>25 years	8	40
	Mean years of experience	24.5	–
Primary area of expertise	Immune cell therapy research and development	6	30
	Regulatory science (industry)	6	30
	Regulatory science (regulatory authorities)	5	25
	Health professions education	3	15

Primary areas of expertise were classified based on participants’ major professional backgrounds and regulatory experience, rather than current institutional affiliation.

### 2.3 Data collection procedure

Data collection began with a literature review in January 2025, followed by subsequent study phases conducted between July and November 2025, in accordance with the four-phase design described in Section 2.1.

#### 2.3.1 Literature review and initial framework development

A multi-source literature review was conducted to identify existing competency frameworks and key competencies relevant to regulatory science and ATMPs. The review covered literature published between 2010 and 2025 and included searches of PubMed and Scopus, as well as regulatory agency websites such as those of the FDA, EMA, the International Council for Harmonisation (ICH), and MFDS. Industry reports and analyses of regulatory science education and training programs were reviewed to capture practice-based perspectives and emerging workforce requirements. Information from these sources was iteratively reviewed, compared, and synthesized to identify competencies. These competencies were then conceptually organized into an initial framework that served as the foundation for subsequent phases.

#### 2.3.2 Preliminary expert consultation

A preliminary expert consultation was conducted to obtain structured feedback on the initial competency framework developed through the literature review. The consultation instrument combined structured rating items assessing the perceived importance and educational relevance of each proposed competency with opportunities for open-ended comments. This design enabled experts to provide both standardized evaluations and qualitative suggestions for refinement. Responses were reviewed descriptively to identify recurring priorities and areas requiring refinement, consolidation, or expansion, rather than analyzed as a standalone quantitative dataset. Findings from this phase informed both the initial refinement of the competency framework and the development of the interview guide for the subsequent semi-structured interviews.

#### 2.3.3 Semi-structured expert interviews

The same 11 experts who participated in the preliminary consultation were subsequently interviewed through semi-structured interviews to enable deeper exploration of competency requirements and refinement needs identified during the consultation. An interview guide was developed based on the literature review and preliminary expert consultation to ensure alignment across phases. Interviews were conducted online or in person, audio-recorded with participants’ consent, and lasted approximately 60–120 min. By the ninth interview, no substantial new conceptual insights had emerged; however, all 11 scheduled interviews were completed to ensure completeness and diversity of perspectives. A detailed interview guide is provided in Supplementary Table S1.

#### 2.3.4 Delphi consensus process

A two-round Delphi survey was conducted with a 20-expert panel to establish expert consensus and refine the competency framework. The Delphi questionnaire was developed based on the competency framework refined through literature review, expert consultation, and semi-structured expert interviews. Delphi questionnaires were distributed individually via email, and panel members were blinded to the responses of other participants.

In Round 1, the competencies and sub-competencies identified in the qualitative phase were evaluated for relevance using a four-point scale (1 = not at all relevant, 4 = highly relevant), clarity using a four-point scale (1 = not at all clear, 4 = very clear), and necessity using a three-category scale for content validity ratio (CVR) assessment (−1 = not necessary, 0 = useful but not essential, and 1 = essential). Qualitative comments regarding potential revisions were also collected. The results were used to refine the competency definitions and improve the overall framework structure.

A summary of Round 1 results, including quantitative findings and major qualitative feedback, was provided to panel members before Round 2 to support reassessment of the revised framework. Consistent with standard Delphi methodology, the same expert panel participated in both rounds to preserve continuity of judgment across iterative refinement (37). Round 2 was conducted approximately seven weeks after Round 1, during which the revised framework was redistributed for further evaluation. Round 2 was designed to confirm the stability of the revised competencies following conceptual refinement by evaluating their importance, educational applicability, and measurability using five-point rating scales (1 = very low, 5 = very high). Panel members also indicated whether each competency should be retained, revised, or removed.

### 2.4 Data analysis

Data from each phase were analyzed using phase-specific methods aligned with the exploratory sequential mixed-methods design. The literature review and preliminary expert consultation informed the identification and refinement of initial competencies and framework elements through an inductive framework-development approach.

Qualitative data from the interviews were analyzed using reflexive thematic analysis following Braun and Clarke's framework (39, 40). Both deductive and inductive coding strategies were employed: an initial coding framework was informed by the preliminary competency structure, while allowing new themes to emerge from the data. The analysis involved familiarization, systematic coding, and iterative development and refinement of themes representing patterns related to the competencies. The qualitative analysis, including coding and theme development, was conducted by the author. Following the analysis, some experts who also participated in the interview and/or Delphi phases were consulted to obtain feedback on the contextual relevance and applicability of the interpreted findings and developing competency framework.

Building on this refined framework, Delphi data were analyzed in accordance with established methodological guidance for achieving expert consensus (41, 42). In Round 1, experts evaluated each competency element for necessity, clarity, and relevance. Necessity was assessed using the content validity ratio (CVR), with a minimum acceptable value of 0.42 for a panel of 20 experts (43). Clarity and relevance were evaluated using descriptive statistics (mean and standard deviation), item-level content validity indices (I-CVI), and degree of agreement. Items that did not meet acceptable levels were revised or removed based on quantitative results and qualitative feedback.

In Round 2, ratings for importance, educational applicability, and measurability were summarized using descriptive statistics and agreement analyses. Experts also indicated whether each competency should be retained, revised, or removed. Retention agreement was calculated as the proportion of experts selecting “retain” or “revise,” whereas “remove” responses were treated as disagreement. Mean ratings for importance (≥4.0), educational applicability (≥3.8), and measurability (≥3.5) were interpreted using study-specific descriptive benchmarks to facilitate comparison across competencies rather than as formal criteria for item retention. Qualitative comments were reviewed to contextualize quantitative findings and support interpretation of the final competency framework.

### 2.5 Ethics

This study was reviewed and approved by the Institutional Review Board of Seoul National University (IRB No. 2508/002-020). All collected data were de-identified, and survey responses and interview data were used solely for research purposes. Written informed consent was obtained from all participants.

## 3 Results

The competency framework was developed through a sequential multi-phase process comprising a literature review, preliminary expert consultation, semi-structured interviews, and a two-round Delphi consensus process. Across these phases, the initial framework derived from the literature review was progressively expanded and refined, culminating in a consensus-based framework comprising nine core competencies and 32 sub-competencies. Each phase contributed distinct evidence that informed iterative refinement of the framework. The overall framework development process is illustrated in Figure 1, and findings from each phase are presented below.

### 3.1 Development of the initial competency framework

#### 3.1.1 Literature-informed competency identification

A multi-source literature review was conducted to identify competencies relevant to regulatory science and ATMPs, particularly in immune cell therapy. The review integrated perspectives from academic research, regulatory policy documents, and industry reports. Academic research informed generalizable competencies, policy documents reflected current regulatory expectations, and industry reports highlighted emerging competency needs associated with advanced therapies.

The synthesis revealed two complementary competency dimensions: (1) general regulatory science competencies applicable across the ATMP product lifecycle and (2) domain-specific competencies reflecting the unique scientific, manufacturing, and regulatory characteristics of immune cell therapies. This distinction informed the dual requirement for foundational regulatory expertise and specialized domain knowledge in emerging therapeutic contexts.

Based on this synthesis, an initial competency framework comprising nine core competencies and 31 sub-competencies was developed. The categorization across competency dimensions is provided in Supplementary Table S2.

#### 3.1.2 Preliminary expert consultation

The preliminary expert consultation highlighted several competency needs not fully reflected in the literature-derived framework. Experts emphasized the need for stronger competencies in chemistry, manufacturing, and controls (CMC), strategic preparation of regulatory submissions, and alignment with international regulatory frameworks. They also identified competency gaps among early-career professionals, particularly in interpreting scientific evidence from a regulatory perspective, constructing scientifically grounded regulatory arguments, developing risk-based regulatory strategies, and integrating multidisciplinary evidence. Collectively, these findings highlighted the importance of competencies extending beyond technical knowledge to include higher-order regulatory reasoning.

Accordingly, the consultation informed the initial refinement of the framework. No competencies were added or removed; instead, refinements focused on enhancing conceptual clarity, reducing conceptual overlap, and strengthening the scientific and regulatory orientation. Several competencies were reframed from task-based descriptions toward competencies emphasizing scientific reasoning and regulatory interpretation. A summary of these refinements is presented in Supplementary Table S3.

### 3.2 Semi-structured expert interview findings: rationale for framework restructuring

Analysis of semi-structured interviews with 11 experts identified four interrelated themes representing key competency dimensions of regulatory science in immune cell therapy. These themes reflect the integrative and adaptive nature of regulatory science in immune cell therapy, shaped by scientific complexity, regulatory uncertainty, and the evolving characteristics of advanced therapies.

#### 3.2.1 Scientific foundations and integrative regulatory reasoning

This theme captures the foundational and cognitive dimensions of regulatory expertise, positioning biological understanding as a prerequisite for evidence interpretation and higher-order regulatory reasoning. Experts emphasized that robust understanding of immune cell biology, underlying mechanisms, and manufacturing variability is essential, given that these living products exhibit dynamic, context-dependent, and often patient-specific characteristics that limit the applicability of fixed regulatory criteria.

“If you don't understand the biology, you can't even interpret the data properly.” (P3, Academia)

“This is not just about checking documents—regulation starts from understanding how these cells actually work.” (P6, Industry)

Regulatory work involves structuring and synthesizing scientific evidence into coherent regulatory arguments, with documentation framed as scientific reasoning rather than administrative output.

“Writing regulatory documents is about structuring scientific evidence logically.” (P1, Academia)

“You have to connect different types of data and make a convincing scientific case.” (P4, Industry)

These findings highlight the integration of biological understanding and scientific reasoning in regulatory decision-making.

#### 3.2.2 Regulatory decision-making under uncertainty and contextual complexity

This theme captures the interpretive and context-dependent nature of regulatory practice in emerging therapeutic domains, where decision-making extends beyond the application of established rules. The absence of fully established regulatory standards requires practitioners to operate under uncertainty, with limited precedents and evolving expectations.

“There is no clear answer in many cases. You have to make decisions without precedent.” (P5, Industry)

“Guidelines exist, but they are not sufficient for these kinds of therapies.” (P8, Regulatory Consultant)

Regulatory work requires the integration of scientific, regulatory, ethical, and contextual considerations rather than the mechanical application of predefined rules. Decision-making often involves navigating incomplete evidence, ambiguous standards, and shifting regulatory expectations. Experts consistently emphasized uncertainty management and contextual interpretation in regulatory decision-making.

#### 3.2.3 Lifecycle-Oriented evaluation and process variability

This theme captures the temporal and process-oriented nature of regulatory expertise, emphasizing evaluation across the entire product lifecycle rather than discrete checkpoints. Continuous changes in process conditions and product attributes require ongoing reinterpretation of evidence.

“The product changes as the process changes, so you have to understand the whole lifecycle.” (P2, Academia)

“CMC is not fixed—you are constantly dealing with variability.” (P7, Industry)

Unlike conventional regulatory contexts, where product characteristics are relatively stable, immune cell therapies involve continuous changes in process conditions and product attributes across development. Experts emphasized the importance of evaluating products across different stages of development. These findings highlighted the importance of competencies related to lifecycle evidence generation, process variability, and adaptive evaluation strategies.

#### 3.2.4 Cross-functional coordination and system-level integration

This theme captures the collaborative and system-level nature of regulatory science in immune cell therapies, highlighting cross-functional coordination as an essential aspect of regulatory science practice. Experts described regulatory science professionals as central coordinators who integrate diverse stakeholders, including scientists, clinicians, manufacturers, and regulators, within complex regulatory environments.

“You are constantly communicating across teams—science, clinical, manufacturing.” (P10, Government/Public Sector)

“It’s a role that connects everything together.” (P11, Government/Public Sector)

Rather than functioning solely as technical specialists, regulatory science professionals emerged as system-level integrators requiring continuous negotiation, alignment, and translation across domains. This coordination extends beyond communication to include the alignment of scientific, clinical, and regulatory perspectives to support coherent, evidence-based regulatory decision-making.

Experts also noted that these integrative competencies are largely acquired through experience because structured educational pathways remain limited.

“There’s no structured training system—you learn everything on the job.” (P9, Regulatory Authority)

“We don’t have enough trained people in this field.” (P6, Industry)

These observations highlight the need for structured educational pathways to support competency development in this field.

#### 3.2.5 Integration of interview findings and refinement of the framework

Taken together, the interview findings indicated that regulatory science decision-making in immune cell therapy is inherently integrative and adaptive. Such decision-making requires context-sensitive integration of scientific knowledge, evolving regulatory frameworks, and professional judgment under conditions of uncertainty and rapid technological change.

“Science and regulation cannot be separated in this field.” (P3, Academia)

“You’re not just applying rules—you’re interpreting science through a regulatory lens.” (P8, Regulatory Consultant)

This integrative and adaptive character served as the central organizing principle underpinning the competency framework. It distinguished regulatory science practice in immune cell therapy from more conventional, rule-based domains and provided the conceptual basis for restructuring the initial framework.

The interview findings informed the refinement and restructuring of the preliminary framework, particularly through the expansion, integration, and reconceptualization of competencies to better reflect the dynamic interplay between biological mechanisms, manufacturing variability, lifecycle considerations, and strategic regulatory judgment. Competencies related to biological knowledge, scientific data integration, and regulatory interpretation were reframed as interdependent components of a unified cognitive process rather than as discrete competencies. Competencies related to lifecycle evaluation, process variability, and decision-making under uncertainty were also strengthened. These refinements resulted in a framework comprising nine core competencies and 36 sub-competencies. The key refinements derived from the interviews are presented in Table 3.

**Table 3:** Summary of framework refinements informed by expert interviews.

Content area	Key refinement	Description
Foundational science & product understanding	Strengthened and deepened	Expanded to emphasize mechanism-based interpretation and biological variability
Regulatory interpretation & strategic judgment	Substantially expanded	Incorporated decision-making under uncertainty and conditions of limited regulatory precedent
Lifecycle-oriented evaluation and process variability	Reframed	Shifted focus toward process variability and regulatory implications across the product lifecycle
Scientific documentation & knowledge integration	Conceptually elevated	Reconceptualized documentation as the structuring and integration of scientific evidence
Cross-functional coordination & regulatory leadership	Expanded	Strengthened competencies related to coordination, cross-functional integration, and regulatory leadership
Global regulatory awareness & adaptive strategy	Integrated	Incorporated cross-jurisdictional regulatory perspectives into strategic decision-making

### 3.3 Delphi round 1: content validity assessment and framework refinement

The restructured framework was subsequently evaluated through the first round of the Delphi consensus process (*n* = 20) to assess the content validity of all proposed competencies.

Overall, most competencies demonstrated strong content validity, with the majority of items meeting the established CVR and I-CVI criteria. Only one core competency (Core Competency 3) did not meet the CVR threshold (CVR=0.40), despite demonstrating high I-CVI and mean scores, indicating strong perceived relevance. Rather than being removed, this competency was conceptually refined based on expert feedback to enhance its distinctiveness and reduce overlap with adjacent competencies before being re-evaluated as part of the revised framework in Round 2. Table 4 presents a summary of the content validity results for the core competencies.

**Table 4:** Content validity results for core competencies: Delphi round 1 (*N* = 20).

Core competency	CVR	*Mean* ± *SD*	I-CVI	Decision
Core 1	1.00	3.95 ± 0.22	1.00	Retained
Core 2	1.00	3.85 ± 0.37	1.00	Retained
Core 3	0.40	3.85 ± 0.37	1.00	Revised
Core 4	0.70	3.95 ± 0.22	1.00	Retained
Core 5	0.60	3.85 ± 0.37	1.00	Retained
Core 6	0.70	3.80 ± 0.41	1.00	Retained
Core 7	0.50	3.75 ± 0.55	0.95	Retained
Core 8	0.50	3.75 ± 0.44	1.00	Retained
Core 9	0.50	3.85 ± 0.60	1.00	Retained

CVR, content validity ratio; I-CVI, item-level content validity index; SD, standard deviation. The minimum acceptable CVR was 0.42 for a panel of 20 experts.

At the sub-competency level, several items showed relatively lower CVR or I-CVI values or were conceptually redundant or insufficiently differentiated from each other. These items were either removed or integrated into related competencies to improve parsimony and conceptual coherence. As a result of this iterative refinement process, four sub-competencies were removed or integrated, and several remaining items were revised, yielding a refined framework consisting of nine core competencies and 32 sub-competencies. The detailed content validity results for all sub-competencies are provided in Supplementary Table S4.

Analysis of the revisions made during Delphi Round 1 revealed three overarching modification patterns. First, redundant or overlapping sub-competencies were removed or integrated to improve parsimony. Second, several competencies were refined to enhance conceptual clarity and strengthen the framework's regulatory relevance. Third, several competency areas were strengthened to more accurately reflect the distinctive characteristics of immune cell therapy regulatory science, including scientific complexity, evolving regulatory standards, and requirements for interdisciplinary integration. Major revisions resulting from Delphi Round 1 are summarized in Table 5.

**Table 5:** Summary of framework revisions following Delphi round 1.

Core competency	Sub-competencies (R1 → R2)	Modification type	Key changes
Core 1	4 → 4	Refinement	Terminology and scope were refined to better reflect mechanism-based scientific interpretation in immune cell therapies
Core 2	4 → 3	Deletion & restructuring	One sub-competency was removed; the focus was shifted toward regulatory interpretation and scientific data evaluation
Core 3	4 → 3	Refinement & integration	The competency was conceptually refined to enhance its distinctiveness from related domains, while redundant sub-competencies were integrated to enhance conceptual clarity and reduce overlap
Core 4	4 → 3	Integration	Overlapping elements were merged to improve parsimony and coherence
Core 5	4 → 4	Refinement	Greater emphasis was placed on the structured integration of scientific and regulatory documentation (e.g., CTD)
Core 6	4 → 4	Expansion	Expanded to incorporate lifecycle-oriented evaluation and benefit–risk considerations
Core 7	4 → 3	Deletion & reallocation	One sub-competency was removed and partially reallocated to strengthen ethical considerations
Core 8	4 → 4	Expansion	Competencies related to interdisciplinary collaboration and global regulatory coordination were strengthened
Core 9	4 → 4	Reorganization	Patient-centered perspectives were restructured and more explicitly integrated within the competency domain

R1, Delphi round 1; R2, Delphi round 2.

### 3.4 Delphi round 2: confirmation of the refined competency framework

The refined framework was evaluated in Round 2 in terms of importance, educational applicability, measurability, and retention agreement. Overall, the revised framework demonstrated consistently high retention agreement across all competencies. A small number of competencies—particularly leadership- and ethics-related sub-competencies—received relatively lower ratings for educational applicability and measurability. However, these items were retained because they were considered conceptually important despite challenges in operationalization. As a result, all core competencies and sub-competencies were retained following Round 2, providing further evidence for the stability of the refined framework.

At the core competency level, all nine core competencies demonstrated 100.0% retention agreement, with mean importance scores ranging from 4.25 to 4.90 and low standard deviations, indicating strong consensus. Core competencies also showed consistently high levels of educational applicability (3.75–4.55) and generally acceptable measurability (3.40–4.30).

At the sub-competency level, retention agreement ranged from 95.0% to 100.0%, with mean importance scores ranging from 3.90 to 4.85, educational applicability from 3.30 to 4.65, and measurability from 3.00 to 4.50. Greater variability was observed at this level, particularly in higher-order competencies such as leadership and ethical responsibility, which received relatively low ratings for educational applicability and measurability. Results for core competencies are presented in Table 6, and detailed results for sub-competencies are provided in Supplementary Table S5.

**Table 6:** Expert rating results for core competencies: Delphi round 2 (*N* = 20).

Core competency	Importance (*Mean* ± *SD*)	Educational applicability (*Mean* ± *SD*)	Measurability (*Mean* ± *SD*)	Retention agreement
1. Immune cell therapy science	4.75 ± 0.64	4.55 ± 0.61	4.20 ± 0.62	100.0%
2. Regulatory science systems and principles	4.90 ± 0.31	4.25 ± 0.64	4.20 ± 0.77	100.0%
3. Regulatory policy intelligence	4.25 ± 0.72	3.80 ± 0.77	3.65 ± 0.88	100.0%
4. Product-centered regulatory strategy	4.70 ± 0.47	4.15 ± 0.75	3.95 ± 0.76	100.0%
5. Regulatory evidence integration and submission design	4.75 ± 0.44	4.55 ± 0.61	4.30 ± 0.47	100.0%
6. Lifecycle evidence and risk management	4.60 ± 0.75	3.85 ± 0.75	3.55 ± 0.83	100.0%
7. Scientific problem solving in regulation	4.70 ± 0.47	3.75 ± 0.91	3.55 ± 0.89	100.0%
8. Multidisciplinary regulatory collaboration and leadership	4.50 ± 0.61	3.85 ± 1.04	3.40 ± 1.10	100.0%
9. Ethics and professional responsibility	4.40 ± 0.75	4.05 ± 0.95	3.50 ± 1.19	100.0%

Retention agreement was defined as the proportion of experts selecting either “retain” or “revise,” whereas “remove” responses were classified as disagreement.

### 3.5 Final competency framework

The final consensus-based competency framework comprised nine core competencies and 32 sub-competencies organized into three interrelated areas: (1) scientific and regulatory foundations, (2) strategic application of regulatory science, and (3) regulatory leadership and responsibility. The complete competency framework, including operational definitions and sub-competencies, is presented in Table 7.

**Table 7:** Final validated competency framework for regulatory science in immune cell therapy.

Competency area & Core competency	Definition	Sub-competencies
Scientific and regulatory foundations		
1. Immune cell therapy science	Understanding the biological mechanisms, technological characteristics, and development processes of immune cell therapies to support informed regulatory evaluation	• Understanding the biological characteristics and mechanisms of immune cells • Interpreting regulatory implications of immune cell therapy technologies • Understanding scientific principles of manufacturing processes • Monitoring technological advances in immune cell therapy
2. Regulatory science systems and principles	Understanding the principles, regulatory frameworks, and systems governing advanced therapy medicinal products and their roles in regulatory decision-making	• Understanding core principles and concepts of regulatory science • Understanding domestic and international regulatory frameworks • Interpreting regulatory documentation and data requirements
3. Regulatory policy intelligence	Analyzing regulatory policies, identifying emerging trends, and anticipating policy changes relevant to immune cell therapy development	• Comparing regulatory policies and requirements across jurisdictions • Interpreting scientific and regulatory implications of policy changes • Assessing regulatory impacts of emerging technologies
Strategic application of regulatory science		
4. Product-centered regulatory strategy	Developing and evaluating regulatory strategies tailored to the characteristics, risks, and development pathways of immune cell therapy products	• Identifying linkages between product characteristics and evaluation outcomes • Planning product-specific regulatory strategies • Validating regulatory strategies through evidence and scenarios
5. Regulatory evidence integration and submission design	Integrating scientific data into coherent and structured regulatory submissions to support regulatory evaluation	• Mapping data to CTD structure and regulatory requirements • Structuring scientific data using regulatory language • Designing stage-specific evidence packages • Ensuring scientific coherence of submission packages
6. Lifecycle evidence and risk management	Managing evidence and regulatory risks across the product lifecycle, from development to post-market surveillance	• Designing lifecycle evidence roadmaps • Conducting benefit–risk assessments • Managing data and regulatory risks across development stages • Monitoring and mitigating lifecycle regulatory risks
Regulatory leadership and responsibility		
7. Scientific problem solving in regulation	Identifying and resolving complex regulatory challenges through scientific reasoning and evidence-based decision-making	• Defining complex regulatory problems • Developing and analyzing regulatory scenarios • Generating scientific justifications for regulatory decisions
8. Multidisciplinary regulatory collaboration and leadership	Facilitating collaboration and leadership across multidisciplinary stakeholders in regulatory processes	• Building multidisciplinary collaborative structures • Designing effective regulatory communication strategies • Leading conflict resolution and negotiation • Promoting international regulatory collaboration
9. Ethics and professional responsibility	Demonstrating ethical integrity, transparency, and accountability while prioritizing patient safety and public health	• Adhering to bioethics and research ethics principles • Demonstrating patient-centered values • Ensuring data transparency and scientific integrity • Engaging in continuous professional development

CTD, common technical document.

## 4 Discussion

This study developed a consensus-based competency framework for regulatory science professionals in immune cell therapy, a field characterized by scientific uncertainty, product variability, and evolving regulatory expectations. Drawing on an iterative mixed-methods development process, the framework comprises nine core competencies organized into three interrelated areas spanning scientific, strategic, and leadership dimensions of regulatory science.

These findings suggest that regulatory competence in advanced therapies is not adequately captured by rule-based or compliance-oriented models but is better understood as a form of evidence-based translational judgment in which heterogeneous scientific evidence is actively interpreted and integrated into context-sensitive regulatory decision-making (44, 45). This interpretation is consistent with prior work on ATMPs, which highlights the limitations of standardized regulatory paradigms given their biological variability, manufacturing complexity, and evolving product attributes (8, 10, 46).

The organization of competencies across these three areas reflects a developmental progression toward adaptive expertise, in which foundational competencies establish the scientific and regulatory schema required for informed regulatory interpretation, whereas higher-order competencies require the integration of multidisciplinary evidence and context-sensitive judgment in complex regulatory environments (47). In this sense, the framework extends CBME beyond traditional clinical settings by illustrating how regulatory reasoning and decision-making develop in complex, rapidly changing professional environments. The findings also reinforce the view that competence is an integrated, context-dependent construct enacted through practice rather than a collection of discrete knowledge or skill components (27, 48).

A notable pattern in the results is the relatively low measurability of higher-order competencies, particularly those related to leadership, professional responsibility, and ethical decision-making. Rather than indicating reduced importance, this pattern reflects a broader measurement paradox within competency-based education, whereby the competencies most critical to complex professional practice are often the most difficult to operationalize and assess (28, 29). Such challenges highlight the limitations of reductionist assessment approaches. Higher-order competencies may therefore require programmatic assessment, workplace-based assessment, and simulation-based assessment strategies that capture performance across time and contexts (49, 50).

The proposed framework differs from existing competency frameworks in regulatory science and health professions education. Unlike prior generalist frameworks, including those developed by RAPS, WHO, and EMA, which primarily enumerate role-based competencies, the present framework embeds domain-specific scientific–regulatory integration and adaptive expertise as organizing principles of professional practice (3, 22, 23). In particular, it positions regulatory professionals as strategic integrators who translate complex and evolving scientific evidence into regulatory action in the context of advanced therapies (46, 47).

From a regulatory science workforce perspective, the proposed framework provides a practical basis for training programs and professional development in advanced therapies. Consistent with CBME principles, the findings suggest that higher-order competencies cannot be effectively developed through didactic instruction alone (27). However, the progression from knowledge integration to strategic and professional competencies necessitates experiential learning approaches that allow learners to navigate uncertainty and complexity. Accordingly, case-based learning, simulation-based learning, and other experiential pedagogies appear particularly well aligned with the framework's developmental logic (51, 52).

Methodologically, this study demonstrates the value of an exploratory sequential mixed-methods design combined with a multidimensional Delphi consensus process, offering a practical blueprint for developing competency frameworks in emerging interdisciplinary fields (41, 53). This approach may be especially useful for other domains in which competency requirements are still evolving and must be defined through iterative expert consensus.

This study had several limitations. The framework was developed within the South Korean regulatory context and may reflect cultural and regulatory specificity. Therefore, international validation involving regulatory agencies and experts from the FDA, EMA, and other regulatory jurisdictions is required to enhance generalizability. In addition, this study focused exclusively on immune cell therapy, and further research is required to examine the applicability of the framework to other advanced therapy medicinal products, including gene and tissue-engineered therapies. Because the study followed a sequential design, competencies evaluated in the Delphi phase were primarily informed by the preceding literature review, preliminary consultation, and qualitative interviews; competencies not identified during these earlier phases may therefore have received less consideration in the subsequent consensus process. Finally, as a consensus-based study, the findings reflect expert judgment rather than actual performance**.** Future research should focus on developing and validating assessment tools and examining the longitudinal relationship between competency development and regulatory outcomes.

## 5 Conclusion

This consensus-based competency framework establishes a foundation for regulatory science workforce development in advanced therapies. These findings highlight the importance of integrating scientific knowledge, regulatory strategy, and professional judgment to support decision-making in complex and evolving regulatory environments.

The proposed framework also offers practical guidance for domain-specific training, competency-based assessment, and professional development in advanced therapies. The findings emphasize the need to move beyond generalist training toward integrated capabilities in scientific reasoning, regulatory judgment, and professional responsibility in real-world regulatory settings. Given the rapid pace of scientific and regulatory change, the framework should be viewed as an evolving foundation that can be refined and extended through future research and application across diverse regulatory settings.

## Data Availability

The datasets presented in this article are not readily available because the datasets are not publicly available due to confidentiality considerations and the potential identifiability of participants. De-identified data are available from the corresponding author upon reasonable request, subject to ethical and institutional requirements. Requests to access the datasets should be directed to Jung-A Lee, jungalee@snu.ac.kr.
