# Supplementary material for: Developing and validating a regulatory science competency framework for immune cell therapy: a mixed-methods study
Source: Front Med (Lausanne). 2026 Sep 16;13:1917978. doi: 10.3389/fmed.2026.1917978 (PMC13623758; doi:10.3389/fmed.2026.1917978)
Supplement: Supplementary file 1 [file Datasheet1.pdf]

Supplementary Material

1 Supplementary Tables

1.1 Supplementary Table S1. Semi-structured expert interview guide

Section	Main questions	Probing questions
**Regulatory context of immune cell therapy**	What are the most distinctive regulatory challenges of immune cell therapy compared to conventional pharmaceuticals?<br>How do product characteristics (e.g., heterogeneity, living cells, and patient-specific variability) influence regulatory decision-making?<br>In which areas are the current regulatory guidelines insufficient or unclear?	Can you provide a recent example of this?<br>How do regulators typically respond to such cases?
**Validation of competency framework**	Which competency domains are the most essential for regulatory professionals?<br>Are any competencies redundant or less relevant?<br>Which important competencies are missing?	Why are these competencies critical?<br>How are these applied in practice?
**Competency gaps and practical needs**	Which competencies are most lacking among early career professionals?<br>What challenges do they face (e.g., IND/BLA, CMC, and communication)?<br>Which competencies differentiate high performers from others?	When do these gaps emerge?<br>How are they addressed?
**Regulatory decision-making and strategy**	How are decisions made when the guidelines are unclear or evolving?<br>What is the role of scientific data interpretation?<br>How important is risk-based thinking and regulatory strategy?	Can you describe a complex decision-making case?<br>What factors most influence the decisions?
**Collaboration and project coordination**	What role do regulatory professionals play in multidisciplinary collaborations?<br>How important is project management?	What challenges arise in terms of coordination?<br>What competencies are required?
**Future-oriented competencies**	Which competencies will become more important in the future?<br>How should regulatory science education evolve in the future?	What changes are driven by these emerging technologies?<br>What should the training prioritize?
**Stakeholder-specific questions**	**Academic experts:** What gaps exist in the current education?<br>**Industry experts:** What challenges arise in IND/BLA preparation?<br>**Regulatory agency experts:** What competencies are essential for reviews?	Academic: How should emerging sciences be integrated?<br>Industry: What determines the success of submissions?<br>Agency: What are the common deficiencies?

Note. IND, Investigational New Drug; BLA, Biologics License Application; CMC, chemistry, manufacturing, and controls.

1.2 Supplementary Table S2. Literature-derived competency domains by conceptual track

Conceptual Track	Core Competency Domain	No. of Sub-competencies
**General Regulatory Science**	Regulatory Knowledge Competency	3
	Scientific Data Interpretation Competency	4
	Information Structuring Competency	3
	Strategic Thinking Competency	4
	Multidisciplinary Collaboration and Communication Competency	3
**Immune Cell Therapy-Specific**	Immune Cell-based Medicinal Products Understanding Competency	3
	Cell Therapy GMP and Quality Management Competency	4
	Immune Cell Therapy Clinical Design and Evaluation Competency	4
**Cross-cutting**	Professional Ethics and Attitude	3

Note. GMP, Good Manufacturing Practice.

1.3 Supplementary Table S3. Framework refinements derived from preliminary expert consultation

Domain	Key refinement	Description of change
Regulatory knowledge	Clarification and extension	Competencies related to regulatory pathway identification and application were strengthened, reflecting the need to address product-specific regulatory routes (e.g., ATMP classification, expedited programs)
Scientific data interpretation	Refinement	Greater emphasis was placed on consistency analysis across IND/BLA documentation and regulatory interpretation of scientific data
Information structuring	Expansion	Competencies related to regulatory information extraction, digital data handling, and cross-functional data integration were expanded
Multidisciplinary collaboration	Expansion	Regulatory project management and coordination across functional teams were incorporated
Professional ethics and responsibility	Consolidation	Ethical competencies were streamlined to focus on professional integrity and consistency

Note. ATMP, advanced therapy medicinal product; IND, Investigational New Drug; BLA, Biologics License Application.

frontiers

1.4 Supplementary Table S4. Content validity results for sub-competencies: Delphi Round 1 (N = 20)

Competency		CVR	Relevance (*Mean* ± *SD*)	I-CVI	Decision
Core 1	Sub 1-1	0.80	3.95 ± 0.22	1.00	Retained
	Sub 1-2	0.70	3.95 ± 0.22	1.00	Retained
	Sub 1-3	1.00	4.00 ± 0.00	1.00	Retained
	Sub 1-4	0.50	3.60 ± 0.60	0.95	Retained
Core 2	Sub 2-1	0.60	3.55 ± 0.83	0.80	Revised
	Sub 2-2	1.00	3.95 ± 0.22	1.00	Retained
	Sub 2-3	0.70	3.75 ± 0.55	0.95	Retained
	Sub 2-4	0.40	3.45 ± 0.89	0.85	Revised
Core 3	Sub 3-1	0.60	3.80 ± 0.41	1.00	Integrated
	Sub 3-2	0.90	3.85 ± 0.37	1.00	Retained
	Sub 3-3	-0.10	3.35 ± 0.81	0.80	Removed
	Sub 3-4	0.50	3.85 ± 0.37	1.00	Retained
Core 4	Sub 4-1	0.90	4.00 ± 0.32	1.00	Revised
	Sub 4-2	0.80	4.00 ± 0.32	1.00	Retained
	Sub 4-3	0.30	3.50 ± 0.83	0.90	Revised
	Sub 4-4	0.40	3.65 ± 0.67	0.90	Retained
Core 5	Sub 5-1	0.70	3.80 ± 0.52	0.95	Retained
	Sub 5-2	0.60	3.70 ± 0.57	0.95	Retained
	Sub 5-3	0.50	3.60 ± 0.60	0.95	Retained
	Sub 5-4	0.70	3.90 ± 0.31	1.00	Retained
Core 6	Sub 6-1	0.70	3.85 ± 0.37	1.00	Retained
	Sub 6-2	0.40	3.65 ± 0.59	0.95	Revised
	Sub 6-3	0.60	3.75 ± 0.44	1.00	Retained
	Sub 6-4	0.30	3.80 ± 0.41	1.00	Revised
Core 7	Sub 7-1	0.40	3.65 ± 0.67	0.90	Revised
	Sub 7-2	0.60	3.75 ± 0.55	0.95	Retained
	Sub 7-3	0.30	3.55 ± 0.83	0.90	Revised
	Sub 7-4	0.30	3.50 ± 0.76	0.85	Revised
Core 8	Sub 8-1	0.60	3.75 ± 0.55	0.95	Retained
	Sub 8-2	0.60	3.80 ± 0.41	1.00	Integrated
	Sub 8-3	0.10	3.55 ± 0.69	0.90	Revised
	Sub 8-4	-0.20	3.40 ± 0.75	0.85	Removed
Core 9	Sub 9-1	0.50	3.95 ± 0.37	1.00	Retained
	Sub 9-2	0.60	3.90 ± 0.22	1.00	Revised
	Sub 9-3	0.00	3.45 ± 0.60	0.95	Revised
	Sub 9-4	0.20	3.75 ± 0.55	0.95	Retained

Note. CVR, content validity ratio; I-CVI, item-level content validity index; SD, standard deviation.

1.5 Supplementary Table S5. Expert rating results for sub-competencies: Delphi Round 2 (N = 20)

Competency		Importance (*Mean* ± *SD*)	Educational Applicability (*Mean* ± *SD*)	Measurability (*Mean* ± *SD*)	Retention agreement
Core 1	Sub 1-1	4.70 ± 0.57	4.55 ± 0.69	4.05 ± 0.76	100.0%
	Sub 1-2	4.50 ± 0.61	4.10 ± 0.97	4.05 ± 0.69	100.0%
	Sub 1-3	4.75 ± 0.55	4.60 ± 0.68	4.30 ± 0.66	100.0%
	Sub 1-4	4.15 ± 0.67	4.20 ± 0.89	3.85 ± 0.93	100.0%
Core 2	Sub 2-1	4.85 ± 0.37	4.15 ± 0.75	3.90 ± 0.91	100.0%
	Sub 2-2	4.85 ± 0.37	4.55 ± 0.51	4.40 ± 0.68	100.0%
	Sub 2-3	4.80 ± 0.41	4.25 ± 0.64	3.95 ± 0.76	100.0%
Core 3	Sub 3-1	4.00 ± 0.92	3.90 ± 0.91	3.70 ± 1.03	95.0%
	Sub 3-2	4.50 ± 0.61	3.90 ± 0.85	3.65 ± 0.88	100.0%
	Sub 3-3	4.35 ± 0.59	3.90 ± 0.85	3.60 ± 0.94	100.0%
Core 4	Sub 4-1	4.75 ± 0.64	4.20 ± 0.89	3.90 ± 0.91	100.0%
	Sub 4-2	4.65 ± 0.67	4.10 ± 0.72	3.80 ± 0.89	100.0%
	Sub 4-3	4.35 ± 0.81	3.90 ± 0.91	3.60 ± 0.94	100.0%
Core 5	Sub 5-1	4.65 ± 0.49	4.50 ± 0.69	4.35 ± 0.59	100.0%
	Sub 5-2	4.30 ± 0.73	4.65 ± 0.49	4.50 ± 0.51	100.0%
	Sub 5-3	4.60 ± 0.68	4.30 ± 0.73	4.15 ± 0.67	100.0%
	Sub 5-4	4.55 ± 0.51	4.20 ± 0.77	4.00 ± 0.73	100.0%
Core 6	Sub 6-1	4.50 ± 0.69	3.80 ± 0.89	3.55 ± 0.95	100.0%
	Sub 6-2	4.35 ± 0.81	3.75 ± 0.85	3.55 ± 0.83	95.0%
	Sub 6-3	4.65 ± 0.59	3.90 ± 0.85	3.75 ± 0.97	100.0%
	Sub 6-4	4.35 ± 0.81	3.55 ± 0.83	3.30 ± 0.98	95.0%
Core 7	Sub 7-1	4.50 ± 0.69	3.90 ± 0.91	3.65 ± 0.88	95.0%
	Sub 7-2	4.55 ± 0.61	3.55 ± 1.00	3.35 ± 1.04	95.0%
	Sub 7-3	4.65 ± 0.59	3.75 ± 0.72	3.40 ± 0.94	100.0%
Core 8	Sub 8-1	4.55 ± 0.69	3.90 ± 0.97	3.50 ± 1.15	100.0%
	Sub 8-2	4.25 ± 0.72	3.85 ± 0.88	3.30 ± 1.03	100.0%
	Sub 8-3	3.90 ± 0.91	3.30 ± 0.98	3.00 ± 1.03	95.0%
	Sub 8-4	4.25 ± 0.64	3.45 ± 1.00	3.10 ± 1.02	100.0%
Core 9	Sub 9-1	4.40 ± 0.75	4.20 ± 0.95	3.55 ± 1.23	100.0%
	Sub 9-2	4.30 ± 0.80	3.90 ± 0.85	3.10 ± 1.17	100.0%
	Sub 9-3	4.60 ± 0.82	3.95 ± 0.83	3.55 ± 1.05	100.0%
	Sub 9-4	4.15 ± 0.81	3.80 ± 1.06	3.45 ± 1.23	100.0%

Note. SD, standard deviation. Retention agreement was defined as the proportion of experts selecting either "retain" or "revise," whereas "remove" responses were classified as disagreement.
